# Some Clinical Forms of Tabes

**Published:** 1910-06-18

**Authors:** F. Raymond

**Affiliations:** Member of the Academy of Medicine; Physician to the Salpetrière Hospital, Paris.


### Hospital Clinics.

### SOME CLINICAL FORMS OF TABES.

(Les formes frustres du tabes.)

From Notes on a Lecture by PROFESSOR F. RAYMOND, Member of the Academy of Medicine; Physician to the Salpetrière Hospital, Paris.

The physician who studies and follows for several years a great number of tabetic cases soon comes to the conclusion that the classic treatises give, on the evolution of tabes, certain indispensable schematic ideas, but that the latter are quite insufficient and incomplete. The symptomatology of tabes is exceedingly rich, and each patient presents a certain number of manifestations forming a particular group with a special physiognomy. No two tabetics are exactly alike. It is particularly in what is called the pre-ataxic period that this polymorphism is bewildering, and as it is very important to be thoroughly acquainted with the different aspects presented by the onset of this affection I shall describe in this lecture three cases in which the diagnosis was rather difficult.

### The First Case.

The first patient is a woman, æt. 37, a dressmaker, who, except for typhoid fever at the age of 13, has always enjoyed good health until the onset of the present trouble. She admits being addicted to slight alcoholic excess. She was working this past winter with a friend, who lives some little distance from her, and one afternoon as she was returning home she suddenly felt a sensation of numbness in the left foot which spread progressively to the whole leg, then to the left side of the body, and finally to the arm, beginning with the hand. In a word, the whole left side except the face became numb. At the same time she experiencd a sensation of intense cold in the same side, felt dizzy, and had to lean against a neighbouring wall in order to prevent herself from falling. Ten minutes later she felt quite well again and walked home. Three days afterwards the very same thing occurred, only this time it was the right side of the body which was affected, and the face and tongue were implicated. For a few minutes she was unable to utter a word, but soon all passed away and she recovered. Four days later a third attack supervened in the left side, but the face remained free.

### Jacksonian Epilepsy.

Since then she has had no further attacks, but whenever she makes a rather sudden movement she feels in her left foot slight tremor and a series of jerks, and lately besides these she has had a few contractions in her left cheek. She therefore decided to come to the hospital for advice. Her general state is good; she has neither headaches nor any sensorial trouble, and no apparent motor symptoms. This woman, gentlemen, is not a neurotic patient; she presents none of the stigmata of hysteria; and if I mention this latter affection, it is only to impress upon you that in the presence of nervous manifestations of any kind whatever it is always necessary to bear in mind the possibility of mere functional trouble.

Admitting, therefore, the organic nature of this patient's malady (and you will appreciate later on the reason for this), there is only one name that we can put to these sensory attacks, starting in the extremity of one of the limbs, spreading to the whole side of the body, and ending by a short suspension of cerebral action, and that is "Jacksonian epilepsy." The term "Jacksonian epilepsy" generally conveys an idea of motor manifestations, but you must know that motor attacks are often preceded or accompanied by sensory phenomena, such as an aching sensation, numbness, or even actual pain. This disease may affect a purely sensory character, and I have reported quite a number of cases; they are more frequently followed by vertigo or by a very slight loss of consciousness than by coma with stertor. We are dealing with one of these cases to-day.

### Some Observations on Pathology.

The pathogeny of this syndrome has not been yet completely elucidated. We know, however, that its cause must be an irritation of the cortex in the sensori-motor zone. Now, in our patient, we do not at first sight perceive the cause of this irritation. There is no history of trauma; it is not a case of cerebral tumour at a fairly advanced stage, at any rate, for there is no headache, no vomiting, no marked vertigo, no ocular symptoms; it is not a case of exogenous intoxication; there is no diabetes, no urinary insufficiency. Apparently, therefore, no objective phenomena can be our guide, and we must make a most minute examination of our patient and see if there are no symptoms which can put us on the track of the initial cause. If the latter is neither mechanical nor toxic, it may be reflex or vasomotor. The patient tells us that she has never had any trouble in walking, and, in fact, a rapid and superficial examination shows that her steps are equal, regular, and that their traces are quite rectilinear. But if we tell her to turn suddenly, or to go down a few steps without looking at her feet, she becomes clumsy and hesitates. Further, she seems at first sight to stand up quite normally, but tell her to close her eyes and you perceive at once a few oscillations which prove that equilibrium is defective. These are symptoms of incipient muscular inco-ordination, and constitute "Romberg's sign."

Muscular force is quite normal in all the limbs, but if, her eyes being shut, we ask her to raise a glass to her lips or to place her left heel on her right knee, in both cases she fails to do so correctly. Both the knee and the Achilles jerks are abolished. The general sensibility appears normal, but the examination of the eye shows two very important things. First, Argyll Robertson's sign, and, secondly, traces of old optic neuritis, consisting in irregularity and a certain degree of discoloration of the optic nerve. There remains one more point, gentlemen, to which I wish to draw your attention: the patient wears a whole set of false teeth. Ask her how she lost her teeth, and she will answer that a few years ago, and in a relatively short space of time, they all got loose and fell out spontaneously without pain or hæmorrhage, and that each tooth appeared to be quite healthy. This dropping out of the teeth is due to trophic trouble, and is comparable to the shedding of the nails and to perforating sore. The diagnosis in this case is therefore perfectly clear; we have ocular symptoms, absence of reflexes, muscular inco-ordination, and these are quite sufficient to declare that our patient is suffering from Duchesne's disease or tabes. There is no real locomotor ataxy yet; she is still in the pre-ataxic period.

### Epileptiform Attacks.

To return for a moment to the sensory attacks this woman had, you are aware that amongst its more unnatural manifestations tabes presents cerebral symptoms, which occur generally during the pre-ataxic period. They consist in apoplectiform or epileptiform attacks, which may present the characters of true epilepsy, of Jacksonian epilepsy, of hemiplegia, or lastly of localised paralysis, with, as Professor Fournier has said, the following threefold characteristics: they disappear; they disappear rapidly; they disappear spontaneously. This very important character (fugacity) shows that they certainly have a vasomotor origin.

At any rate, these symptoms give the disease the aspect of a cerebral affection, and make it differ very considerably from the ordinary medullary type of tabes. Further, the insidious onset of these symptoms may very well cause the diagnosis to incline rather to some ischæmic or inflammatory disease of the brain, cerebral syphilis, etc. etc.

There is another point of interest to note. Among the symptoms of the prodromal period of general paralysis of the insane one of the most important consists in sudden epileptiform attacks resembling either true epilepsy or localised epilepsy. The latter may be either motor or sensory in character, but the sensory variety is particularly significative, and may be considered as one of the most characteristic prodromata of general paralysis. Now, in our patient, besides the sensory attacks we find certain symptoms which on a superficial examination might escape notice. Her character is rather irregular and changeable, there is slight tremor of the tongue, and slight mumbling in her speech. These symptoms, however trifling they may appear, must cause the prognosis to be reserved. If this woman were, in the near future, to develop general paralysis, I should not be at all surprised.

### The Second Case.

The second patient is a strong-looking man of 39, a carrier by profession. He came to the hospital on account of chronic and recurring ulcerations on the soles of both feet. They began eight years ago; an ulceration, or as we may call it straight away, a perforating sore, appeared on the little toe of the right foot, ran on to suppuration, and ended in sloughing of two phalanges. Three years later a second ulceration appeared on the big toe of the left foot, and a third on the plantar aspect of the metatarso-phalangeal joint of the same digit. A fourth soon developed on the little toe of the left foot, followed by suppuration and sloughing of the third phalanx. A fifth appeared on the big toe of the right foot, which had to be amputated three years later. Finally, during the past year a sixth sore has appeared on the outside border of the fifth left metatarsal bone. Let us sum up the present state of things:—Left foot: A perforating sore in evolution; the loss of substance is circular, with clean-cut edges; the floor is irregular and discharges a little purulent serosity. There is no inflammatory reaction round the ulceration, and there is complete anæsthesia of the latter. A director may be introduced right down to the bone, and even into the joint, without producing the slightest pain. We further notice the complete elimination of the third phalanx of the little toe and the scars left by the two sores on the big toe. Right foot: Big toe amputated; little toe reduced to a fleshy, purple mass; traces of small sores on the second and third toes. Both feet are cold, purple coloured, and the skin is macerated by excessive perspiration. There is a certain degree of œdema of the left foot; a papular eruption on the outer side of the leg and brown pigmentation on the inner side.

### The Pathology of Perforating Ulcer.

There have been many theories concerning the pathogeny of perforating sore. I do not intend to discuss them here, and will simply confine myself to saying that it is the neuritic theory which is generally accepted to-day, and which adapts itself to all the causes—nervous, toxic, or diathetic—which preside over the development of the sore. Undoubtedly prolonged local pressure may play the part of occasional cause, but the real cause of the trophic trouble must be looked for elsewhere. First of all, it is the group of diseases of the cord which must engage our attention, particularly tabes, then anterior polyomyelitis, Friedreich's disease, the different varieties of myelitis, and syringomyelia. A perforating sore may be caused by trophic trouble due to pressure on the cord (Pott's disease, tumour) or to spina bifida. We have sometimes to go further afield and look for the cause in diffuse meningo-encephalitis, even in the absence of all lesion of the posterior tracks, as Barthelemy has shown. More frequently the cause is in a primary or secondary affection of the peripheral nerves—wounds, contusions, sections, pressure, neuritis of infectious or toxic nature, etc. etc.

The ætiology of perforating ulcer may be of a more general nature. For example, some infection, such as leprosy; some intoxication, such as alcohol or mercury; some diathesis, such as diabetes; some dystrophy, such as arterio-sclerosis. All these causes act on the cutaneous tissues in two ways: first, by giving rise to a state of malnutrition of these parts due to faulty circulation and to greater toxicity of the blood; secondly, indirectly by causing trophic alterations of the nerves. The soles of the feet and the toes are not the only localisations of the lesions; these may also be found in the palm of the hand, in the sacral and coccygeal regions, and Dr. Henry described in 1906 cases of perforating sores in the mouth.

If we question our patient, we find that he had, at the age of 17 or 18, a chancre, followed in due course by roseola and mucous patches. He underwent repeated courses of mercurial treatment, and only a little while ago he was given injections of calomel for tertiary manifestations of the tongue, where we can still perceive traces of leucoplakia.

Quite spontaneously he tells us that towards the age of 25 he suffered a good deal from violent pains in the legs, consisting in sharp tearing and dragging sensations coming on and ceasing suddenly. In spite of these characters they were considered to be of a rheumatic nature. This is a mistake which occurs daily, and one against which I especially want to put you on your guard. These pains disappeared in the course of a few years, and were followed by girdle pains and a very unpleasant sensation of constriction in the chest. During the last few years they have very much improved, and the patient has only now and then a few slight pains with formication and numbness in the lower extremities.

### Lightning Pains.

The description of these pains immediately draws our attention to the idea of locomotor ataxy. Examination of the knee jerks show that although they are not completely abolished they are markedly diminished. At first sight the patient does not seem to be ataxic. He walks quite freely, he turns round easily and rapidly when told to do so, he has no difficulty in going up or down stairs. If, however, he shuts his eyes a certain degree of inco-ordination appears; he presents a little hesitation when he begins walking, and also when he stops; he throws his legs forward rather jerkily. When standing, he totters and finds it difficult to keep his equilibrium. Muscular force is quite normal, but the muscles of the legs and arms present a certain degree of flaccidity, and the limbs can be flexed to a greater extent than is the ordinary case; there is hypotonia. The patient says that during the last few months he has had some difficulty in micturition; he has often to make a considerable effort for the first few drops of urine to appear, and sometimes he has to assume the squatting position in order to pass water. Now and then he has had a little incontinence of urine, and more rarely of the fæces.

### Some Further Initial Symptoms.

Sexual desire is diminished, and testicular sensibility is also diminished. Examination of the eye shows no lesion of the fundus nor any trouble of the external muscles, but there is marked irregularity of the pupils and diminished reaction to light.

The above, gentlemen, are the symptoms which may be found in our patient; they had to be looked for, and might easily have been passed over at a superficial examination. This explains how the correct diagnosis was not made before, although the disease is of 14 years' standing. Apart from the trophic trouble for which the patient came to the hospital, I wish to draw your attention to the following four important symptoms: pain, Rombergs' sign, urinary trouble, and the ocular symptoms. Any three of these are sufficient for the diagnosis of tabes; the adjunction of the fourth makes it certain. The reflexes exist, it is true, but, however important their presence may be, it is not an absolute objection to the diagnosis of locomotor ataxy.

### Some Trophic Disturbances.

In this patient, where the defects in the gait and equilibrium are not noticeable unless specially looked for, the course of the disease has already lasted 14 years and is still at the pre-ataxic period. The latter has been very protracted, but may still last for some time. What particularises this case are the numerous sores, which have destroyed two toes and opened a joint. This constitutes the trophic form of tabes in which the nutrition of the lower extremities is thoroughly disorganised.

I wish you also to notice that both feet present a marked impairment of the plantar arch. This deformity, as you are aware, may in a great many cases be put down to tabes itself; it is here due to trophic trouble having given rise to weakening of the joints and relaxing of the fibrous tissues.

There are certain accessory causes which explain the special form of the disease in this patient. He is very tall, has very long feet, and from boyhood has had to walk and stand a great deal. He is a varicose subject, and it seems his father suffered from arterio-sclerosis. All these together constitute a predisposition to nutritive trouble of the lower extremities. It is no wonder, therefore, that the neuritic lesions of the disease were very rapidly followed by the series of trophic symptoms.

Similar cases are not so rare as one might imagine, and you must bear in mind that trophic trouble is not only a symptom of confirmed tabes but may supervene at the very outset of the disease. Do not think, therefore, that the examination of the reflexes constitutes the criterion of the diagnosis.

### A Third Case.

The third patient is a woman, æt. 33; married at 15, divorced and remarried at 28; has had one child, who died aged two months. She had some uterine trouble after her confinement, probably due to infection, but she is not able to give any very definite information about it. Pain and a chronic discharge, however, persisted after it, and four years ago the Fallopian tubes were removed. As she presented at that time attacks of vomiting (you will understand the reason for this later), the surgeon removed the appendix also.

The operation cured the genital trouble, but the vomiting persisted. About six years ago she suffered from violent pains in the legs, and, quite spontaneously, she describes them as "knife thrusts" or "lightning pains." They used to come on suddenly every day about one hour after going to bed, would last half an hour or so and then disappear. These pains lasted altogether two or three months. As usual, and I cannot insist too much upon this point, they were considered to be of a rheumatic nature, and treated as such without any improvement ensuing.

Persistent Vomiting.

Very soon the vomiting became more frequent, the matter brought up being of a bilious nature. The attacks came on at any moment of the day; they were quite independent of the meals, and change of diet had no influence on them. They were more frequent during the menstrual period, but note carefully that they were absolutely painless. The vomiting continued with these characters during four years, when they presented a new aspect. Instead of the attacks being quite painless as before, they were now accompanied by violent pains in the back, in the walls of the chest, and in the epigastric region. The pains were sometimes of a perforating character, as in ulcer of the stomach; at others of a sharp shooting sensation, like a belt round the body; and sometimes the patient experienced a sensation of constriction as if pressed in a vice. The attacks were frequent, and, as before, more so during menstruation. Each attack lasted at first two or three days, then eight to fifteen days, and the patient in order to prevent the pain appearing avoided all food. Now and then diarrhœa came on after the attacks, followed by considerable loss of weight, and the patient would then get into a very serious state of nervous depression. She has tried all kinds of remedies to calm the pain; many have had no effect whatever, the only one which has had a sedative action being morphine. This drug she has taken to excess, and I will tell you a little later on the part it may have played in creating the frequency of the attacks. The pains in the legs had never quite disappeared, and, in fact, she had pains in the upper extremities also, affecting especially the ulnar aspect of the forearm and the little finger.

During a period of five years these various pains constituted the only manifestation of the disease, but during the last year or so she noticed that if she did not pay special attention when walking she would trip over the slightest obstacle and that she had some difficulty in going down steps. Now and then she is affected with diplopia, and her husband has noticed that she has then slight strabismus. From time to time she passes urine, and sometimes fæces, unconsciously.

The Diagnostic Points.

I have now given you the history of the case. You have, of course, guessed the diagnosis, and a rapid examination confirms it. She has marked inco-ordination when walking with the eyes shut, and throws her legs forward in a characteristic fashion. Romberg's sign is present; the Achilles jerks are absent, and the knee jerks are diminished; the examination of the eyes shows the presence of Argyll Robertson's sign. There is not the slightest doubt that the patient is suffering from tabes, and the diagnosis is quite easy, but the point is: Was it so a few years ago? All physicians know that gastric symptoms may exist during the pre-ataxic period, but what they do not realise so much is that these symptoms may be the first to usher in the disease. How often have they not been considered as due to gastric ulcer, to gastritis with intermittent pyloric stenosis, to hepatic or nephritic colic? The student has always in his mind the complete picture of the ordinary classic tabetic attack; he does not sufficiently bear in mind its great variety of forms, its incomplete manifestations. This patient has had periodically attacks of painless vomiting. Another may have attacks of gastralgia without any vomiting. These different manifestations have only one characteristic in common, and that is their periodicity.

A Note on Treatment.

There is another point I wish you to note, namely, the influence on the frequency of the attacks of dyspepsia due to errors in diet, or to gastritis caused by the use of sedative drugs. This may give rise in the intervals of the real tabetic paroxysms to attacks less acute and less prolonged. I need not remind you of the disastrous effects of frequent and repeated injections of morphine on the stomach, this organ being one of the important means of elimination of the drug. The more frequent the injections, the more frequent and the more prolonged are the gastric paroxysms. This is what has happened in our patient, and moreover it is certain that morphine has played no small part in causing the marked nervous depression from which she suffered some months ago. And now what is to be done for this patient? Well, the first thing to do is to cease the regular administration of morphine and to allow its use only when the pains are extremely violent. We must then put her on a diet—milk only during a few days, and then a lacto-vegetarian diet.

Assuaging the Pains.

In order to calm the pains the best method which we have at our disposition, to my mind, is the injection of a solution of nitrite of soda. I generally follow the system of Petrone in Italy, and of Pal and Winternitz in Vienna. Every day during 10 consecutive days I inject one cubic centimetre of a 2 per cent. solution of nitrite of soda; I then allow the patient to rest for 10 days. The next 10 days 2 c.c. each day, followed by 10 days' rest; another series with 3 c.c., then a rest; and finally 4 c.c. of the solution, *i.e.* 8 centigrammes of nitrite of soda, during the following 10 days.

I have obtained very good results with the above technique, although I must say that I have had failures also. Liebermann's method is being experimented in the laboratory of the Clinique Charcot. It consists in ultra-violet ray baths applied to the lumbar region for a quarter of an hour three times a week. It is too early yet to form an opinion as to its therapeutic value.

I have not attempted in this lecture to present to you any new clinical anatomical facts; my desire has been simply to show that in tabes, more perhaps than in any other affection, you must expect to encounter abnormal and incomplete forms.

---

Next week's clinic will be by W. H. Clayton-Greene, M.B., F.R.C.S., on "The Treatment of Certain Fractures."

